# Pulmonary Dissemination in Adult‐Onset Recurrent Respiratory Papillomatosis: A Rare and Severe Manifestation

**DOI:** 10.1002/rcr2.70375

**Published:** 2025-10-07

**Authors:** Abhir Nainani, Chui Lyn Leong, Samar Ojaimi, Ignatius Rudd, Albert Yin, Paul Paddle, Simon Knight, Tracy Leong

**Affiliations:** ^1^ Department of Respiratory and Sleep Medicine Austin Health Melbourne Australia; ^2^ Immunology Laboratory Monash Health Melbourne Australia; ^3^ Department of Pathology Austin Health Melbourne Australia; ^4^ Department of Otolaryngology, Head and Neck Surgery Monash Health Melbourne Australia; ^5^ Department of Thoracic Surgery Austin Health Melbourne Australia

**Keywords:** HPV, lung cavitation, malignancy, papillomatosis

## Abstract

Recurrent respiratory papillomatosis (RRP) commonly affects the upper airways, and lung parenchymal involvement is extremely rare. We present a case of adult‐onset laryngotracheal RRP with lung parenchymal involvement that progressed despite systemic bevacizumab and ultimately required a left lower lobectomy. Histological features were characteristic of a squamous papilloma. Unusually, parenchymal involvement was present, with solid intra‐alveolar nests cradled by preserved alveolar septa. Cytologic atypia and keratinisation were present; however, the absence of stromal desmoplasia and invasion precluded a diagnosis of squamous cell carcinoma and made overall classification challenging in this case. This case highlights the complexity of managing RRP with pulmonary involvement, particularly when disease progression persists despite systemic therapy such as systemic bevacizumab.

## Graphical abstract

Recurrent respiratory papillomatosis (RRP) commonly affects the upper airways, and lung parenchymal involvement is extremely rare. We present a case of adult‐onset laryngotracheal RRP with confirmed distal dissemination that progressed despite systemic bevacizumab, ultimately requiring surgical resection. This case highlights the complexity of managing RRP with pulmonary involvement, particularly when disease progression persists despite systemic therapy with limited efficacious options, highlighting the need for more robust clinical trials.

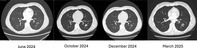

## 1 Introduction

Recurrent respiratory papillomatosis (RRP) is characterised by benign tumours in the respiratory tract due to chronic human papillomavirus (HPV) infection, most commonly types 6 and 11 [1]. Adult‐onset RRP, which is usually localised to the larynx, is less common and aggressive, while pulmonary distal dissemination is extremely rare and associated with a worse prognosis [1].

The variability in symptoms such as cough, dyspnoea, wheeze and recurrent pneumonia can lead to delays in diagnosis in RRP [1]. Computer Tomography (CT) may reveal airway narrowing, polypoid nodules or cavitating lesions, but definitive diagnosis requires bronchoscopy, biopsy and detection of HPV. Approximately 4% of patients with RRP undergo malignant transformation into bronchogenic squamous cell carcinoma (SCC), with higher rates in those with pulmonary involvement [1].

There is no cure for RRP, and management focuses on maintaining airway patency and voice through surgical excision. Adjuvant systemic therapies have been explored as management strategies in aggressive cases to modify disease progression; however, there are no randomised trials to guide their use [1, 2].

We present a case of adult‐onset laryngotracheal RRP with lung parenchymal involvement that progressed despite systemic bevacizumab and ultimately required a left lower lobectomy.

## 2 Case Report

A 56‐year‐old female, immunocompetent, lifelong non‐smoker presented with dyspnoea. She had a 20‐year history of HPV‐11 RRP, localised to the larynx and mid‐trachea, complicated by supraglottic stenosis. This was symptomatically managed with two to three monthly microlaryngoscopy, laser debridement, intra‐lesional bevacizumab injections and supraglottic dilatations. She received the Gardasil9 HPV vaccine.

A CT chest revealed a 13 mm lobulated lesion in the left lower lobe. Radial endobronchial ultrasound showed erythema in her left bronchus, but the lesion itself was not visualised. Endobronchial brushings and biopsies were benign, with negative cultures and no HPV detected on PCR.

**FIGURE 1:**
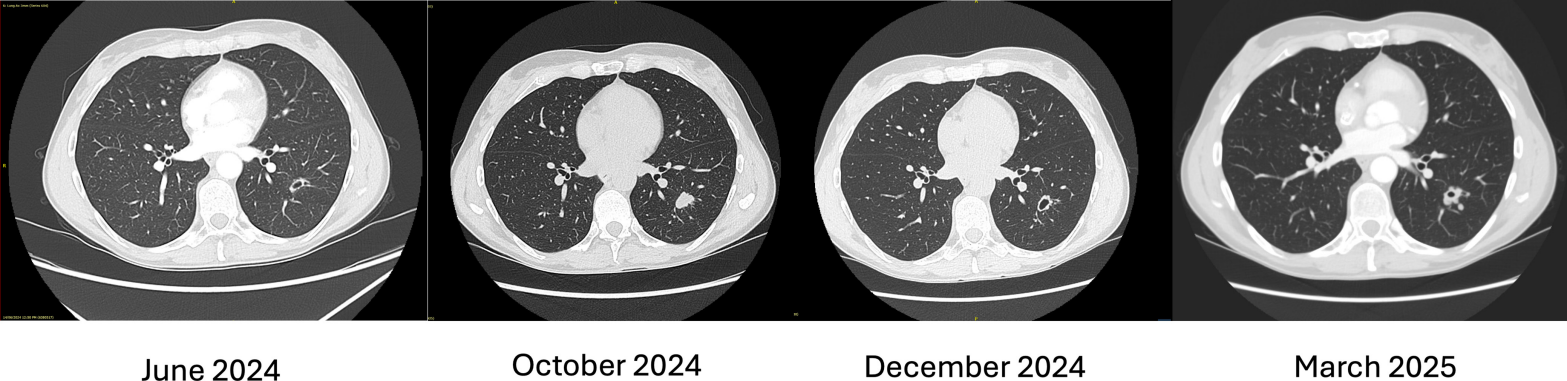
Serial CT Chest imaging taken June 2024, October 2024, December 2024 and March 2025.

The lesion was consistent with bronchial papilloma, given the RRP history, although mild FDG‐avidity raised concerns for malignancy. She was started on 640 mg of systemic bevacizumab. Despite completing five doses, serial CT scans over a year demonstrated progressive lesion cavitation, new pulmonary nodules bilaterally and persistent upper airway disease requiring interval surgical interventions (Figure 1).

Tissue sampled on a repeat radial endobronchial ultrasound adjacent to the lesion was positive for HPV‐11 on PCR. Six further doses of bevacizumab were administered. Subsequent imaging showed central opacification of the cavitated lesion and interval growth of adjacent nodules, with no nodal or distant disease on a repeat PET scan. Due to concern for malignant transformation, she proceeded to video‐assisted thoracoscopic left lower lobectomy.

**FIGURE 2:**
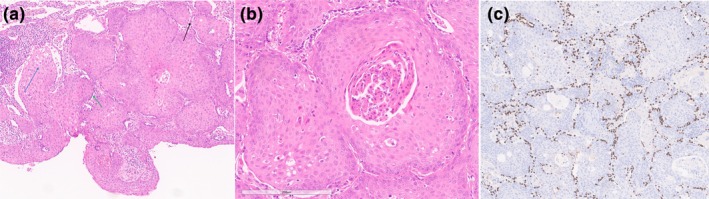
Histology. (a) Low‐power image (4× magnification): Bronchial papilloma with intra‐alveolar growth pattern (blue arrow), preserved alveolar septa (black arrow) and hyperplasia of entrapped alveolar pneumocytes (green arrow). (b) High‐power image (20× magnification): Mild cytological atypia with keratinisation. (c) TTF‐1 immunohistochemistry highlighting alveolar pneumocytes along preserved septa confirming an intra‐alveolar growth pattern.

Histological features were characteristic of a squamous papilloma. Unusually, parenchymal involvement was present, with solid intra‐alveolar nests cradled by preserved alveolar septa. Cytologic atypia and keratinisation were present, however, the absence of stromal desmoplasia and invasion precluded a diagnosis of SCC (Figure 2). p53 immunohistochemistry showed a wild‐type pattern and the Ki67 index was 40%–50%, predominantly highlighting the basal layer, overall consistent with low‐grade dysplasia.

The patient remains asymptomatic approximately 3 months post‐surgery and is planned for surveillance CT imaging in 1 month.

## 3 Discussion

Pulmonary dissemination occurs in approximately 1% of RRP cases. While the exact mechanism remains unclear, theories include contiguous spread, hematogenous dissemination and aspiration of infected particles [1, 3]. Our case supports the aspiration hypothesis, as papillomas were confined to the laryngotracheal and smaller airways with no direct extension to the bronchi. In contrast, contiguous spread would likely have led to sequential involvement from the larynx to the bronchi, while hematogenous dissemination would have resulted in scattered lesions throughout the respiratory tract. Risk factors for distal spread include rapid progression, tracheal involvement, early onset and frequent endoscopic interventions (e.g., laryngoscopy), which may cause fragmentation, aerolisation and aspiration of infected tissue to the distal airways through passive gravitational spread and inhalation [3].

Pulmonary lesions in RRP can present as cysts, masses, nodules or cavitations [4]. In our case, the pulmonary lesion evolved, initially enlarging, then cavitating, solidifying and fluctuating in size. Lesion evolution was influenced by various factors including intermittent airway obstruction, inflammation and treatment effects. This variability complicates the distinction between benign papillomas and malignant lesions as both can be FDG‐avid on PET scans [3].

Dysplastic lesions in RRP are caused by HPV integration into host DNA, leading to TP53 protein degradation and increased p16 protein. P16 acts as a marker for HPV integration, but it was negative in our case, making diagnosis challenging [5]. High‐grade dysplasia, especially with risk factors such as smoking, immunosuppression or high‐risk HPV subtypes carries a significantly higher risk [5]. However, no standardised histopathological grading system or diagnostic pathway exists to guide prognosis, management and surveillance. This is relevant to our patient who underwent over 10 CT scans in 2 years leading to cumulative radiation exposure. While radiation‐limiting modalities such as low‐dose CT or MRI have been proposed, they remain poorly evaluated in RRP. MRI is further limited by cost, accessibility and reduced sensitivity for detecting small or thin‐walled lesions [1]. With increasing efforts to detect malignant transformation early [4], there is a growing need for surveillance strategies that balance radiation exposure, logistical constraints and accurate identification of clinically significant lesions.

Several adjuvant non‐surgical therapies are available including cidofovir which inhibits HPV DNA replication but is associated with nephrotoxicity, bone marrow suppression, localised fibrosis and possible malignant transformation. Interferon may reduce recurrence but carries substantial systemic toxicity [2]. Bevacizumab, an anti‐VEGF, has shown encouraging results in severe cases, with reduced surgical intervention in 95% of patients being treated with systemic bevacizumab and complete surgical avoidance in 56% with either intralesional or systemic bevacizumab [2]. Importantly, bevacizumab is well‐tolerated, with hypertension and proteinuria being the most common adverse effects. Emerging therapies targeting the PD‐1 pathway have shown potential however, their efficacy, particularly in treating pulmonary lesions, appears to be limited, highlighting the importance of more robust clinical trials [2].

In conclusion, this case highlights the complexity of managing RRP with pulmonary involvement, particularly when disease progression persists despite systemic therapy and emphasises the need for ongoing research into standardised diagnostic pathways, histopathological grading systems and effective treatments for patients with severe disease. In our patient, these challenges are compounded by uncertainty around optimal surveillance intervals and strategies to prevent further distal spread, particularly in the context of recurrent upper airway obstruction requiring ongoing surgical intervention.

## Author Contributions

S.O., P.P., S.K. and T.L. were directly involved in this patient's care. I.R. and A.Y. were indirectly involved in this patient's care. C.L.L., A.N. and T.L. conceptualised the project. A.N. and C.L.L. authored the manuscript. C.L.L., S.O., I.R., A.Y., P.P., S.K. and T.L. reviewed the manuscript. Authors are from different institutions, as this patient required multispecialty care from various health networks in Melbourne, Australia.

## Consent

The authors declare that written informed consent was obtained for the publication of this manuscript and accompanying images using the consent form provided by the Journal.

## Conflicts of Interest

T.L. is an Editorial Board member of Respirology Case Reports and a co‐author of this article. She was excluded from all editorial decision‐making related to the acceptance of this article for publication. The other authors declare no conflicts of interest.

## Data Availability

The data that support the findings of this study are available on request from the corresponding author. The data are not publicly available due to privacy or ethical restrictions.
